# Risky Play and Children’s Safety: Balancing Priorities for Optimal Child Development

**DOI:** 10.3390/ijerph9093134

**Published:** 2012-08-30

**Authors:** Mariana Brussoni, Lise L. Olsen, Ian Pike, David A. Sleet

**Affiliations:** 1 Department of Pediatrics, School of Population and Public Health, British Columbia Injury Research and Prevention Unit, Child and Family Research Institute, University of British Columbia, British Columbia Children’s Hospital, L408-4480 Oak Street, Vancouver, V6H 3V4 BC, Canada; 2 British Columbia Injury Research and Prevention Unit, Child and Family Research Institute, L408-4480 Oak Street, Vancouver, V6H 3V4 BC, Canada; Email: lolsen@cw.bc.ca; 3 Department of Pediatrics, British Columbia Injury Research and Prevention Unit, Child and Family Research Institute University of British Columbia, British Columbia Children’s Hospital, L408-4480 Oak Street, Vancouver, V6H 3V4 BC, Canada; Email: ipike@cw.bc.ca; 4 Centers for Disease Control and Prevention, National Center for Injury Prevention and Control, 4770 Buford Highway NE F-62, Atlanta, GA 30341, USA; Email: dds6@cdc.gov

**Keywords:** injury prevention, outdoor play, risky play, active play, child development, playground safety

## Abstract

Injury prevention plays a key role in keeping children safe, but emerging research suggests that imposing too many restrictions on children’s outdoor risky play hinders their development. We explore the relationship between child development, play, and conceptions of risk taking with the aim of informing child injury prevention. Generational trends indicate children’s diminishing engagement in outdoor play is influenced by parental and societal concerns. We outline the importance of play as a necessary ingredient for healthy child development and review the evidence for arguments supporting the need for outdoor risky play, including: (1) children have a natural propensity towards risky play; and, (2) keeping children safe involves letting them take and manage risks. Literature from many disciplines supports the notion that safety efforts should be balanced with opportunities for child development through outdoor risky play. New avenues for investigation and action are emerging seeking optimal strategies for keeping children “as safe as necessary,” not “as safe as possible.” This paradigm shift represents a potential for epistemological growth as well as cross-disciplinary collaboration to foster optimal child development while preserving children’s safety.

## 1. Introduction

Unintentional injuries are a leading cause of death and hospitalization for children worldwide, taking the lives of nearly a million children each year [1]. As deaths from communicable diseases are decreasing, injuries remain a leading cause of childhood mortality and morbidity and represent an increasingly significant public health burden [2]. The grim statistics that on average 720 Canadian [3], over 12,000 American children [4] and 42,000 European children [5] ages 0–19 die every year from injuries make the need for injury prevention abundantly clear. 

Injury prevention plays a key role in promoting children’s safety, which is considered to involve keeping children free from the occurrence or risk of injury. However, emerging research suggests that imposing too many restrictions on children’s outdoor risky play may be hampering their development. Like safety, play is deemed so critical to child development and their physical and mental health that it is included in Article 31 of the United Nations Convention on the Rights of the Child. Thus, limitations on children’s play opportunities may be fundamentally hindering their health and well-being. Eager and Little [6] describe a risk deprived child as more prone to problems such as obesity, mental health concerns, lack of independence, and a decrease in learning, perception and judgment skills, created when risk is removed from play and restrictions are too high. Findings from disciplines such as psychology, sociology, landscape architecture and leisure studies, challenge the notion that child safety is paramount and that efforts to optimize child safety in all circumstances is the best approach for child development [7,8,9,10]. Furthermore, parents, popular culture, the media, and researchers in other disciplines have expressed views that child safety efforts promote overprotection of children [9,11]. For example, numerous media stories have suggested that excessive health and safety regulations and overprotective parenting practices serve to diminish children’s outdoor risky play opportunities [12,13,14]. Likewise, numerous parenting books disparagingly describe parents perceived to be excessively curtailing their children’s independence and voluntary physical risk taking opportunities [15,16,17,18]. These have the potential to trigger a backlash against proven safety promotion strategies, such as child safety seats or necessary supervision [19], possibly reversing the significant gains that have been made in reducing child injuries [20,21]. In short, it is timely and important to reflect on our approach towards safety with respect to children’s outdoor risky play opportunities and to consider the impact on healthy child development. 

In this paper, we explore from an interdisciplinary, public health oriented perspective, the relationship between child development, play, and conceptions of risk taking relating to outdoor risky play. Our aim is to contribute to the discussion on whether the goal of unintentional physical injury prevention should be to keep children as safe as possible or as safe as necessary. We conclude with recommendations for child play safety efforts based on key empirical and theoretical findings. 

## 2. What is Free Play?

No matter how you define play, it is a dominant activity of children’s daily life in all cultures [22]. The study of play has been truly interdisciplinary, yielding research literature from nearly every field affecting human health and well being. By definition, free play is intrinsically motivated and not provoked by instrumental goal-directed behaviour [23]. It is a goal in itself and lacks external rules and structure [23]. Thus, activities such as organized sports would not be considered free play. 

Three main types of free play have been well described in the literature: physical activity play (e.g., exercise play, rough-and-tumble play); object play (e.g., manipulating objects, toys); and pretend play (e.g., socio-dramatic) [24]. Risky play is subsumed within physical activity play and has been defined as thrilling and exciting and where there is a risk of physical injury [25]. Sandseter [26] further categorizes risky play into play involving: heights, speed, dangerous tools, or near dangerous elements (e.g., fall into something), and where children can get lost. The focus of this paper is on free play with specific attention to risky play occurring in the outdoors. 

### Why is Free Play Important?

Children’s free play has been recognized as a major agent in young children’s development and learning [27]. Through play, children learn societal roles, norms, and values and develop physical and cognitive competencies, creativity, self-worth and efficacy [24,28]. Play has been described as the work of children which helps them develop intrinsic interests, learn how to make decisions, problem-solve, exert self-control, follow rules, regulate emotions, and develop and maintain peer relationships [23]. Risk taking in play helps children test their physical limits, develop their perceptual-motor capacity, and learn to avoid and adjust to dangerous environments and activities [29]. Play is biologically based and provides an evolutionary contribution to human development and changes [22]. Furthermore, children also report being happiest when at play [30]. 

A U.S. longitudinal study provides compelling evidence of the importance of free play on healthy development [31]. Sixty-eight disadvantaged children were randomly assigned to participate in one of three preschool curricula at ages 3 to 4 years. Two of the classes included at least 21% free play and child-initiated activity component. The third class focused on direct instruction of academic skills and allowed for only 2% of free play activities. When tested at age 15, children in the latter class were significantly more likely than the other classes to experience misconduct, and less likely to participate in active sports or contribute to their family or community. Furthermore, at age 23, problems worsened with significantly higher levels of work suspensions and arrests [31]. These findings underline that free play is fundamental to healthy child development, and that restriction of free play in the preschool years might potentially have lifelong repercussions. 

Animal research provides further evidence of the impact of early free play on healthy development. Rats isolated during the two weeks of development when they most frequently play (weeks 4 and 5) displayed subsequent social disturbances [32]. Additional research with rats indicated that plentiful free play opportunities facilitated maturation of the frontal lobe and thus executive functioning [33,34]. In children, these brain areas house regulatory skills, which help control impulsive urges and promote self-reflection, creativity and empathy. 

Outdoor free play, especially in versatile natural environments, is important for developing motor fitness and abilities, environmental awareness and navigation competencies, as well as promoting creativity [24,35]. A Scandinavian study comparing an experimental group of 46 children aged 5 to 7 years in a kindergarten with ready access to a natural play environment with 29 children from two kindergartens with access to traditional outdoor playgrounds found that children in the experimental group used the environment to play in more versatile ways and, nine months later, showed significantly greater improvement in various measures of motor ability [36]. The studies described above and many others [37] strongly support abundant opportunities for outdoor free play as a basic and essential need for healthy child development.

## 3. Declining Opportunities for Outdoor Free Play

There is considerable evidence that children’s opportunities to engage in outdoor free play and their geographic freedom have steadily declined across the past several generations [38,39,40,41]. A recent U.S. study reported almost half of preschool children were not being taken outside to play by a parent on a daily basis [42]; and while 70% of U.S. mothers reported daily outdoor free play when they were children, only 31% reported that their own children did the same [43]. One study of U.S. childhood experiences from 1915 to 1976 showed substantial decreases in children’s free play and access to their neighbourhoods beginning in the 1940s and declining steadily over time [44].

Children’s ready access to computers and televisions has contributed to an increased proportion of their leisure time being spent indoors, both historically and as children age; and outdoor free play activities are being substituted with organized sporting or other activities such as music lessons [10]. U.S. reports showed a 37% decline in participation in outdoor activities for children aged 6 to 12 years from 1997 to 2003 [39]. Furthermore, in one study 59% of children aged 5 to 12 years preferred indoor play to any other location [45]. In Canada, a study of 878 children aged 6 to 11 years wearing accelerometers indicated 7.6 hours of sedentary time per day [46]. Their parent reported an average of 2.5 hours/day of this was screen time. In a survey of 51,922 Canadian children and youth in Grades 6 to 12 (aged approximately 11–18 years), they self-reported an average of 7.8 hours of screen time daily [47]. These studies suggest that children and adolescents increase their sedentary behaviour and screen time as they age. By all accounts, children’s leisure lives appear to be moving indoors. 

The changes in children’s play habits and subsequent effects on their physical and mental health are concerning. Childhood obesity rates have steadily increased, which have been linked to a decrease in physical activity. In 1978–1979, 15% of Canadian children were considered overweight or obese [48]. The figure rose to 26% in 2004 and continues to climb. In the U.S., childhood obesity rates have tripled in 30 years [49]. In 2008, 30% of children were considered overweight or obese [50]. Research in Europe also indicates increasing paediatric obesity [51], with current estimates for 6 to 9 year olds at 24% [52]. Research has indicated the importance of encouraging free play as an antidote to rising obesity and as a means to increase daily physical activity [53].

A recent review documents the decline of outdoor play and the rise of psychopathology in children and adolescents suggesting a causal link [54]. Gray [54] argues that play deprivation can contribute to a reduced sense of personal control, reduced ability to control emotions, increased social isolation, and reduced happiness; all of which are associated with anxiety and/or depression. Indeed, anxiety and depression scores on standardised measurement tools have increased sharply since the 1950’s, with current generations of young people having five to eight times more clinically significant scores [55,56].

### Why Outdoor Free Play is Declining

Societal influences on parents have been cited as important drivers of changes in children’s outdoor play opportunities. Increased societal concerns about child safety have heightened parental concerns, especially with regard to traffic dangers and child abduction by strangers [10,43,45,57]. For example, in a U.K. study of 1,011 parents, 43% believed that children under the age of 14 years should not be allowed outside unsupervised, and half of those parents felt they should not be allowed outside unsupervised until they were 16 years of age [58]. Parents’ perceptions about danger can be disproportionate to actual dangers. While traffic concerns are borne out by statistics, child abductions by strangers are exceedingly rare. Ironically, “stranger-danger” concerns have resulted in increasing volumes of traffic, with corresponding increases in traffic-related dangers [57].

Furthermore, current Western middle class social pressures to maximize children’s opportunities and adhere to practices of “intensive parenting” support the notion that parents should have children attend the “best” schools, participate in a multitude of organized activities, and provide as much protection as possible—potentially more than they personally perceive as necessary [10,57,59,60,61]. The result has been creation of a “backseat generation” with little unstructured play time and reliance on automobile-based commuting from one activity to the next [38]. 

In an Australian study asking 50 children aged 4 to 8 years old to photograph their daily activities, half of the children included photographs of being driven [59]. Additionally in a U.K. study, girls from middle class backgrounds displayed less knowledge about their neighbourhoods than girls from less affluent backgrounds, because more of their leisure time was spent indoors or in supervised activities, which could affect their ability to negotiate public space safely [10]. These changes in exposure to opportunities to interact with the environment can deprive children of the opportunity to learn important survival skills that only experience with risk can provide. The goal should not be to eliminate all risks, but to control risk, and teach children and adolescents how best to manage risks [62]. 

Recent decades have seen changes in societal perceptions of children’s competencies and resilience [61]. From once considering children as actively responsible and capable, we have more recently moved to viewing them as inadequate by comparison to adults, leading to a perception that children need to be protected from their own inadequacies [10,63]. These trends have contributed to placing limits on children’s exploration and access to outdoor free play opportunities [10,64]. For example, stemming from their study of children’s use of playspaces in 16 childcare centres, Herrington and Nicholls [9] argued that the Canadian Standards Association’s own standards for children’s playspaces and equipment did not reflect children’s developmental and play needs, but rather the goals of risk reduction. 

In a similar study examining the play preferences and playground equipment usage of Australian boys and girls ages 48 to 64 months, the authors noted children’s preference for equipment and activities that allowed them to experience the sensations of height and speed such as slides, swings, monkey bars and climbing ropes [8]. They also noted that the equipment at the five parks studied provided few opportunities for building on or mastery of existing skills, or for learning new skills. They concluded that the equipment provided reflected the priorities of local government (with perhaps a strong focus on safety) rather than those of the children and their preferences for risky play [8]. Jambor [65] noted the concern that insufficient challenge can easily lead to boredom, potentially promoting inappropriate equipment use and excessive risk taking behaviour that is often associated with unintentional injury.

The result of parental and societal fears and influences has been a decline in play spaces offering children opportunities to test out their competencies and imagination. This is despite indications that fears are at odds with trends showing steady decreases in injury rates [20,21] and the relative rarity of playground-related deaths. For example, Ball [7] estimated the odds of playground-related death in the U.K. as less than 1 in 30 million children *per annum* for children aged 0 to 16 years. While we do not advocate dangerous play environments, we believe it is appropriate to consider how to optimize play opportunities to support children’s developmental needs while considering safety. 

## 4. Support for Outdoor Risky Play

Research indicates that children have a need for outdoor risky play opportunities. Two main bodies of evidence are reviewed below. 

### 4.1. Children Have a Natural Propensity towards Outdoor Risky Play

Undoubtedly, some children have greater appetite for risks than others [66,67]. However, children’s propensity for some degree of risky play appears to be universal [25,37]. Naturalistic observations of preschool children engaging in outdoor free play indicate deliberate exposures to risk, such as playing at heights and high speeds [68]. The author notes that children appeared to understand their personal competencies and the level of risk they were comfortable with and moderated their risky play to these internal boundaries. They also understood and accepted that peers would have different levels of comfort and ability. 

A study of Australian children ages 48 to 64 months that collected observational and interview data on 38 children indicated that when provided with a choice 74% of participants preferred to play on the more challenging playground equipment. Furthermore, while only 21% to 34% of children had experience using the higher risk equipment (e.g., flying fox, space net, tubular slide), 70% to 90% expressed the desire to play on this type of equipment [8]. While there may be the need to use caution in interpreting these results, as children’s stated desires may not equate to behaviours, Morrongiello [69] has found a correlation between children’s willingness to engage in risk taking behaviours and actual risk taking behaviours.

Animal research demonstrates the propensity for risk taking during play across species. For example, primates at play deliberately expose themselves to moderately frightening situations where they repeatedly lose and regain control of bodily movements [70]. During play fighting, rats prefer the riskier, more physically and emotionally challenging subordinate position [71]. 

Research suggests that if children perceive they are not obtaining challenging and interesting risky play opportunities in public play areas, they may seek these opportunities elsewhere. A survey of 1,973 children aged 11 to 14 years in a deprived area of England indicated that over 40% regularly visited and played in wastelands, building sites, underpasses, rivers, abandoned buildings and quarries [72]. These children were also more likely to have sustained an injury in the previous month. The surveyed children overwhelmingly reported wanting their local area to be made safer and have more interesting things to do, suggesting that they recognized the danger and risk of injury of their chosen play spaces but had little choice of available and desirable play spaces. 

There is evidence to support concerns that absence of opportunities for outdoor risky play will result in children disengaging from physical activity. One Canadian study documenting preschool children’s use of play equipment in 16 childcare centres found that play equipment was used only 13% of the time and was used as intended only 3% of the time [73]. U.S. childcare providers in one study expressed concerns that overly strict standards had rendered outdoor play areas unchallenging and uninteresting to children, thus hampering their physical activity [74]. Furthermore, participants noted that some children used equipment in unsafe ways to maintain challenge. 

### 4.2. Keeping Children Safe Involves Letting Them Take and Manage Risks

Parental concerns regarding children’s safety have been shown to be the most significant influence on children’s access to independent play [40,45]. Research has found that parents recognize that their early restrictions on children’s play has the potential for putting their children at increased risk once they gain more independence [10,11]. Numerous studies indicate that children want to be trusted with decisions with respect to managing risks and safety [10,75,76]. In one U.K. study, focus groups were conducted with 93 children aged 7 to 11 years and living in urban and rural areas. Results showed that the children felt strongly about being afforded opportunities for assessing risk for themselves [75]. They created identities reflecting maturity and competence and which included being able to display their ability to manage risks. Taking risks allowed them to display courage and physical skills to themselves and their peers. Interestingly, while they viewed minor injuries as a way to show that risks had been taken, there was an understanding that too many injuries indicated carelessness or clumsiness, which was perceived in derogatory ways [75]. Thus, they appeared to have their own regulatory system for maintaining risks and injuries at a manageable level. 

Children in other U.K. research [10] perceived themselves as competent at negotiating their own safety. Furthermore, they felt that they, and not their parents, were primarily responsible for their own safety. In many cases the children had more detailed knowledge of the local area than their parents and used it to negotiate spaces safely. 

There is evidence that children learn risk management strategies for themselves and their peers as a result of risky play experiences. Observational studies of children at play found they exposed themselves to risk but displayed clear strategies for mitigating harm [68,76]. Australian children, for the most part, engaged in behaviours that were well within their current capabilities [8]. Children appeared aware of potential dangers and adjusted activities accordingly. Notably, children drew on their risk experiences not only to develop understanding of their own constitutions and skills, but also of playmates. These understandings facilitated support for each other’s risk engagement and safety [76]. 

Sandseter and Kennair [25] theorized that children’s engagement in risky play has an adaptive function in reducing fear of stimuli (e.g., heights) through repeatedly naturally and progressively exposing themselves to the stimuli. They argued that if children were not provided with sufficient risky play opportunities, they will not experience their ability to cope with fear-inducing situations. Furthermore, they will maintain their fear, which may translate into anxiety disorders. Support for these argument also comes from animal research, which has shown that young rhesus monkeys and rats deprived of play during critical development points later show excessive fear, inappropriate aggression and exaggerated emotional reactions in stressful situations [71,77]. Importantly, anxiety disorders are the most prevalent mental disorder in children and adolescents and parental overprotection has been associated with increased rates [78]. 

## 5. Alternative Free Play Environments that Manage Risks

“Adventure playgrounds” may be a potential solution to providing safe play environments that afford opportunities for risk taking [79]. First established in 1943 by Danish landscape architect Sørensen after observing children’s play in construction sites and junkyards, he sought to provide children with dedicated space to foster otherwise prohibited play [80,81]. Adventure playgrounds were subsequently championed in England during the postwar era in reaction to the lack of interest children showed to conventional playgrounds and in seeking to provide creative spaces appealing to boys and girls of all ages [80]. They emerged as staffed and unstaffed play spaces where play workers could provide supervision and assistance, while still giving children the freedom to pursue their own interests. Adventure playgrounds provide child-centered and child-directed play spaces where children create and modify their own environments [80]. Children have access to raw materials such as building supplies and tools, as well as sand, dirt and water. In some cases, adventure playgrounds include trained play workers and volunteers for supervision and “professional scaffolding” that facilitates children’s play and removes play barriers [81]. Different opportunities exist for children of varying developmental levels and interests to try new things, such as climbing that is graded for developmental requirements, allowing children to select risk they are comfortable with. Some adventure playgrounds in proximity to farms or community gardens provide children with the opportunity to interact with and care for animals, and grow and cook their own food [81]. While there are estimates of approximately 1,000 adventure playgrounds in Europe, they have not been widespread in North America, which is believed to be the result of culture-specific safety concerns [81]. 

Research on adventure playgrounds, safety and child development is in its infancy and few academic peer-reviewed articles are available. There are accounts in the grey literature indicating lower injury rates than conventional playgrounds [81], reductions in aggressive behaviour and gains in social responsibility and social problem solving [82]. Organizations such as Play England [79] are exploring methods for promoting playground settings and adventure playgrounds that do not have the same cost implications of staffed adventure playgrounds, yet manage injury risk. Their guide describes how to undertake a risk-benefit assessment to determine the benefits and risks of a play area and activity focusing on “hazards with the potential to cause real harm” and incorporating considerations with respect to local circumstances and needs [79]. Clearly further investigation is required to understand the developmental and safety implications of adventure playgrounds. However, early data are promising and encourage serious consideration of this model in promoting child risky play.

## 6. Conclusions

Children’s need for play has been globally recognized as a basic childhood right. Numerous developmental and health advantages have also been linked to children’s need for outdoor risky play as a means to learn through experience. Societal trends limiting children’s access to outdoor risky play opportunities combined with a culturally dominant excessive focus on safety can pose a threat to healthy child development. Eager and Little [6] have coined the term “Risk Deficit Disorder” to describe a set of problems that children can experience resulting from attempts to remove risk from their lives. Our examination of the evidence would suggest that such a label is premature. However, we share their concerns with respect to the trends evident in aspects of child safety efforts relating to outdoor play. 

We would encourage the injury prevention field to foster opportunities to engage in outdoor risky play that align with safety efforts. An approach can be encouraged that focuses on eliminating hazards, which Wallach [83] (as cited in [65]) defines as a source of harm that is not obvious to the child, such that the potential for injury is hidden, such as a broken railing; but does not eliminate all risks, which involve a situation that allows the child to recognize and evaluate the challenge and decide on a course of action that is not dangerous, but may still involve an element of risk. This approach has been advocated elsewhere [84] and is a central component of the Adventure Playground movement. Notably, European and Australian organizations and researchers appear to be attempting to operationalise this idea in practice, with North American efforts lagging. For example, the National Institute for Health and Clinical Excellence in the U.K. released injury prevention guidelines that called for policies that counter “excessive risk aversion” and promote children’s need “to develop skills to assess and manage risks, according to their age and ability” [85]. Both injury and play organizations, such as the U.K.’s Royal Society for the Prevention of Accidents [62] and Play Safety Forum [84] promote the idea of keeping children *as safe as necessary, not as safe as possible*. International collaboration would benefit from translating this into practice in a manner that is sensitive to concerns for child safety and children’s developmental needs for risky play. 

Research is emerging which considers optimal strategies for providing children with outdoor risky play opportunities that minimize hazards, such as adventure playgrounds [6,79,86] or provision of unstructured play materials that can be freely manipulated in conventional playgrounds [73,87]. These novel areas of investigation have the potential to open up many exciting avenues for injury prevention and represent an opportunity for epistemological growth, cross-disciplinary and international collaboration to foster optimal child development.
